# High performance polymer solar cells with as-prepared zirconium acetylacetonate film as cathode buffer layer

**DOI:** 10.1038/srep04691

**Published:** 2014-04-15

**Authors:** Zhan'ao Tan, Shusheng Li, Fuzhi Wang, Deping Qian, Jun Lin, Jianhui Hou, Yongfang Li

**Affiliations:** 1 State Key Laboratory of Alternate Electrical Power System with Renewable Energy Sources, Beijing Key Laboratory of Energy Security and Clean Utilization, North China Electric Power University, Beijing 102206, China; 2 Beijing National Laboratory for Molecular Sciences, Institute of Chemistry, Chinese Academy of Sciences, Beijing 100190, China

## Abstract

Low-work-function active metals are commonly used as cathode in polymer solar cells (PSCs), but sensitivity of the active metals towards moisture and oxygen results in poor stability of the devices. Therefore, solution-proceessable and stable cathode buffer layer is of great importance for the application of PSCs. Here we demonstrate high performance PSCs by employing as-prepared zirconium acetylacetonate (a-ZrAcac) film spin-cast from its ethanol solution as cathode buffer layer. The PSCs based on a low bandgap polymer PBDTBDD as donor and PC_60_BM as acceptor with a-ZrAcac/Al cathode demonstrated an average power conversion efficiency (PCE) of 8.75% which is significantly improved than that of the devices with traditional Ca/Al cathode. The improved photovoltaic performance is benefitted from the decreased series resistance and enhanced light harvest of the PSCs with the a-ZrAcac/Al cathode. The results indicate that a-ZrAcac is a promising high performance cathode buffer layer for fabricating large area flexible PSCs.

Since the first reports of photo-induced electron transfer from a conjugated polymer to fullerene^1^, polymer solar cells (PSCs) have attracted exclusive attention due to low-cost, light-weight, and mechanical flexibility with compatibility to future large-area roll-to-roll printing production. The performance of PSCs depends not only on the electronic energy levels, absorption and carrier mobility of the conjugated polymer donor and fullerene derivative acceptor photovoltaic materials^2^,^3^,^4^,^5^, but also on the effective charge extraction of both holes and electrons from the photoactive layer and then collection to the respective anode and cathode^6^,^7^,^8^,^9^. Therefore, the electrode materials or the electrode buffer layers play a key role in improving photovoltaic performance of the PSCs^7^,^8^. High workfunction anode buffer layers and low workfunction cathode buffer layers are pursued in selecting the electrode buffer layers.

Low-work-function active metals, such as Ca, Ba and Mg, are commonly used as cathode for efficient electron extraction in PSCs, but the active metals are very sensitive to environmental moisture and oxygen, resulting in poor stability of the devices^10^,^11^,^12^,^13^,^14^. Therefore, solution-proceessable and stable cathode buffer layer (CBL) is of great importance for promoting the application of PSCs^15^,^16^,^17^. Recently, solution processable transition metal oxides (ZnO^18^,^19^,^20^,^21^ and TiOx^22^,^23^,^24^,^25^), transition metal chelates^26^,^27^,^28^,^29^,^30^, and conjugated polyelectrolytes^31^,^32^,^33^, have been successfully used as the CBLs. Nevertheless, compared with anode buffer layer, the CBL is still very limited, and air-stable, facile-processed, easily obtained CBLs are in great request. Recently, a wide bandgap oxide, zirconium oxide (ZrO_2_), deposited by spray pyrolysis^34^, electron beam evaporation^35^ or atomic layer deposition^36^, was successfully utilized as electron injection layer in inverse and conventional polymer light-emitting diodes. The greatly enhancement in device performance was attributed to the suitable energy levels of ZrO_2_ which have hole-blocking and electron injection ability^34^,^35^,^36^. However, there is no report about using zirconium based materials as CBLs in PSCs.

In this work, we demonstrate high performance PSCs by employing as-prepared zirconium acetylacetonate film (a-ZrAcac) as CBL. The a-ZrAcac CBL was simply prepared by spin-coating its ethanol solution on photoactive layer at room temperature, no thermal annealing or any other post-treatment was performed. To investigate the photovoltaic performance of the a-ZrAcac CBL, bulk-heterojunction PSCs based on P3HT (poly(3-hexylthiophene)) or low bandgap D-A copolymer PBT1 (poly(1,3-bis(2-ethylhexyl)-5,7-bis(4-(2-ethylhexyl)thiophen-2-yl)benzo[1,2-c:4,5-c′]dithiophene-4,8-dione)-co-(2,2′-bithiophene)), PBDTTT-C-T (poly(4,8-bis(5-(2-ethylhexyl)-thiophene-2-yl)-benzo[1,2-b:4,5-b′]dithiophene-alt -alkylcarbonyl-thieno[3,4-b]thiophene)), and PBDTBDD (poly(((4,8-Bis(5-(2-ethylhexyl)thiophen-2-yl)benzo[1,2-b:4,5-b′]dithiophene-2,6-diyl) bis(trimethyl))-co-(5,7-bis(2-ethylhexyl)benzo[1,2-c:4,5-c′]dithiophene-4,8-dione))) as electron donor and PC_60_BM or PC_70_BM as electron acceptor were fabricated with a-ZrAcac/Al as cathode. The molecular structures of donor, accepter and ZrAcac are shown in Figure 1(a). The power conversion efficiency (PCE) of the P3HT:PC_60_BM-based device with a-ZrAcac CBL reaches 4.23%, which is nearly 60% increased in comparison with the PSC without the buffer layer and ca. 12% increased than that of the PSC with traditional Ca/Al cathode. For the PSCs with low bandgap polymer PBDTBDD as electron donor, an average PCE of 8.75% with a maximum of 9.23% was achieved with a-ZrAcac CBL, greatly improved in comparison to the devices with Al (5.72%) or Ca/Al (7.34%) as cathode.

## Results

The a-ZrAcac layer is highly transparent in the visible wavelength range as shown in Figure S1 in Supporting Information (SI) and bearing an amorphous structure confirmed by XRD (Figure S2 in SI). The characteristic absorption peak of acetylacetonate located at 300 nm attributes to the n-π* and π-π* intra-ligand electronic transitions^37^,^38^. The transparence of the a-ZrAcac layer will benefit the transmission and reflection on the back Al electrode for the transmitted light through the active layer, which will potentially increase the light harvest of the photoactive layer and thus enhance the photocurrent of the PSCs. a-ZrAcac possesses a high stability constant^39^ and is thermally stable at lower than 150°C as shown in the thermogravimetric analysis (TGA) and differential thermal analysis (DTA) plots in Figure S3 in SI. Our result is in good agreement with previous study^40^.

**Figure 1:**
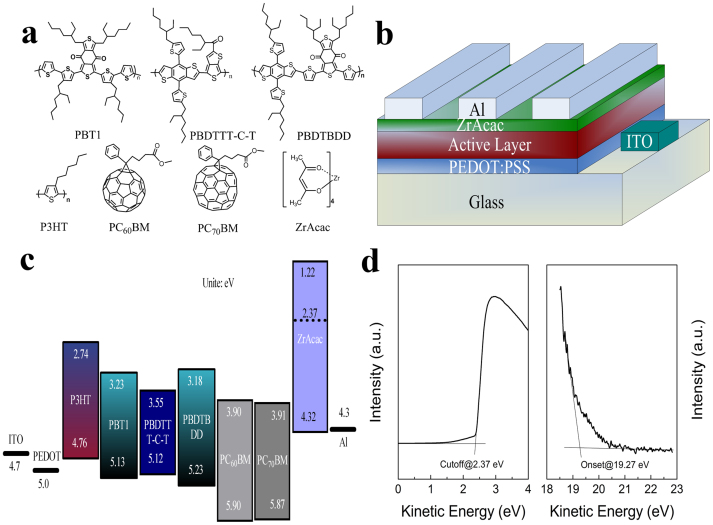
(a) Molecular structures of P3HT, PBT1, PBDTBDD-C-T, PBDTBDD, PC_60_BM, PC_70_BM and ZrAcac; (b) Device structure of the polymer solar cells; (c) Schematic energy diagram of the materials involved in the PSCs; (d) UPS spectra of a-ZrAcac on ITO substrate.

The effects of a-ZrAcac CBL on the photovoltaic performance of PSCs were examined by constructing the traditional structured devices of ITO/PEDOT:PSS/Active layer/a-ZrAcac/Al, as shown in Figure 1(b). In the PSCs, the active layer is P3HT:PC_60_BM, P3HT:PC_70_BM, PBT1:PC_70_BM, PBDTTT-C-T:PC_70_BM or PBDTBDD:PC_60_BM blend film, PEDOT:PSS is poly(3,4-ethylenedioxythiophene):poly(styrenesulfonate). Figure 1(c) shows the highest occupied molecular orbital (HOMO) and the lowest unoccupied molecular orbital (LUMO) energy levels of P3HT^41^, PBDTBDD^42^, PBT1^43^, PBDTTT-C-T^44^, PC_60_BM^45^, PC_70_BM^46^ and a-ZrAcac, where the energy levels of ZrAcac were measured by ultraviolet photoelectron spectroscopy (UPS). Figure 1(d) illustrates the UPS spectra taken from the ZrAcac coated on ITO substrate. The work function can be deduced from the energy of the secondary cutoff, which is 2.37 eV. The HOMO level can be calculated as 4.32 eV from the difference between the incident light energy (He I, 21.22 eV) and the energy of the onset (19.27 eV) as well as the work function. The LUMO level of 1.22 eV was obtained from the band gap of 3.1 eV (determined from onset of the absorption (400 nm) (see Figure S1 in SI)) and the HOMO level. The energy level alignment at the photoactive blend layer/a-ZrAcac interface is very important for high performance PSCs. The extremely low work function of ZrAcac (2.37 eV) lies above the LUMO energy levels of PC_60_BM (3.90 eV) and PC_70_BM (3.91 eV), inducing the interfacial dipole formation across this interface, which will facilitate “barrier-free” electron extraction from the LUMOs of PC_60_BM and PC_70_BM^47^. The good energy level matching of ZrAcac can expect excellent electron extraction from photoactive layer and enhanced performance of the PSCs.

**Figure 2:**
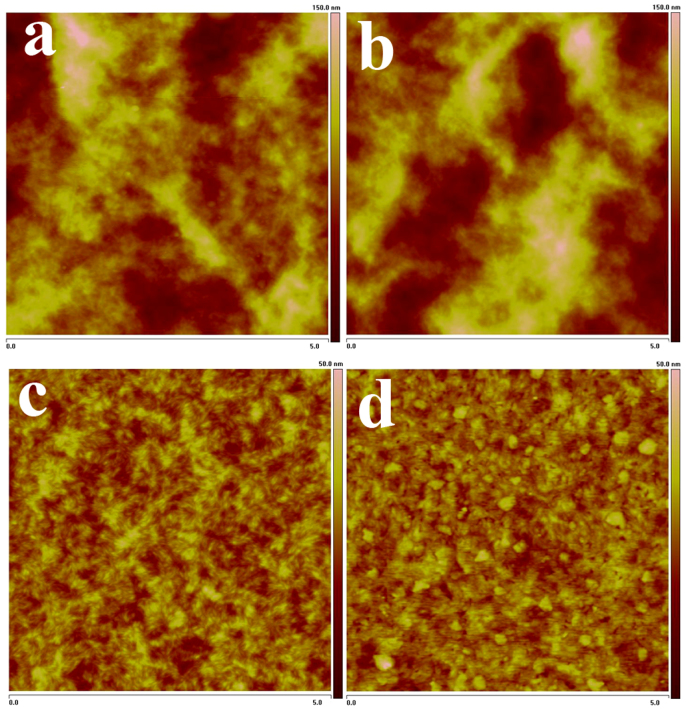
AFM images of P3HT:PC_60_BM blend film on ITO/PEDOT:PSS substrate without (a) and with (b) a-ZrAcac layer, and AFM images of PBDTBDD:PC_60_BM blend film on ITO/PEDOT:PSS substrate without (c) and with (d) a-ZrAcac layer. The scan size is 5 μm × 5 μm.

Surface morphology of a-ZrAcac layer spin-coated on clean ITO glass surface was investigated by tapping-mode atomic force microscopy (AFM), as shown in Figure S4 in SI. The AFM image shows a root-mean-square (rms) roughness of 3.4 nm, which is a little lower than that (4.6 nm) of bare ITO surface as shown in Figure S5. The surface morphologies of the photoactive layer with and without a-ZrAcac CBL were also investigated by AFM, as illustrated in Figure 2. The rms roughness of the blend layer of P3HT:PC_60_BM is 16.9 nm (Figure 2(a)), while the surface covered by the a-ZrAcac layer becomes more rough with a rms of 19.6 nm (Figure 2(b)). The same roughness changing trend but smaller roughness was observed for the blend layers of PBDTBDD:PC_60_BM. The rms roughness of the blend layers of PBDTBDD:PC_60_BM with and without a-ZrAcac CBL is 5.5 nm (Figure 2(d)) and 2.7 nm (Figure 2(c)), respectively. The increased roughness for the active layers with a-ZrAcac CBL could increase the contact area with the Al electrode deposited on it^48^.

**Figure 3:**
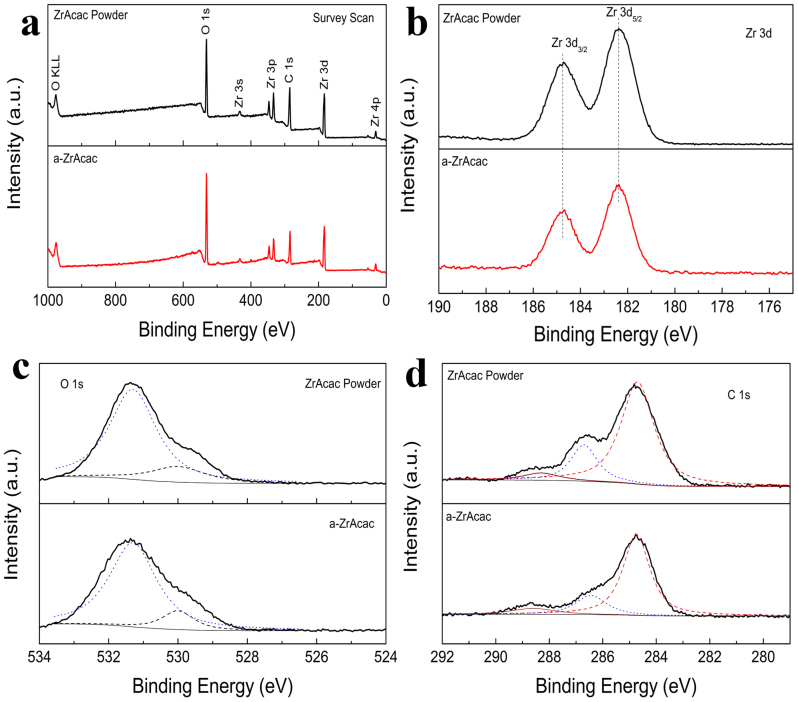
X-ray photoelectron spectroscopy (XPS) of ZrAcac powder and a-ZrAcac film (40 nm) on glass substrate. (a) survey scan, (b) Zr 3d, (c) O 1s, and (d) C 1s core level spectra.

The chemical component of ZrAcac powder and as prepared Zracac layer (a-ZrAcac, 40 nm) on glass slide was analyzed by X-ray photoelectron spectroscopy (XPS) as shown in Figure 3. The binding energies (BE) obtained in the XPS analysis are corrected for specimen charge by referencing the C 1 s peak to 284.8 eV. As shown in Figure 3(a), the survey scan for both powder and thin film samples performed in the 0–1000 eV BE range show characteristic peaks of the elements Zr, O, and C. As shown in Figure 3(b), the XPS spectrum of the core level of Zr 3d for the powder sample has a strong spin–orbit doublet due to Zr 3d_5/2_ at 182.3 eV and Zr 3d_3/2_ at 184.7 eV^49^,^50^. The XPS spectrum is constrained by the Zr 3d_5/2_–Zr 3d_3/2_ spin–orbit separation being 2.4 eV, and the area ratio of the two peaks of each doublet being 3:2. These features are characteristic of Zr^4+^ ions in full oxidation states^49^,^50^. In the XPS spectrum of the a-ZrAcac thin film sample, the peaks corresponding to Zr 3d5/2 and Zr 3d3/2 are unchanged at 182.3 eV and 184.7 eV, respectively, with an Zr 3d5/2–Zr 3d3/2 spin–orbit separation of 2.4 eV and an intensity ratio of 3:2. The results indicate that the zirconium in the thin film sample is also in the Zr^4+^ oxidation state. The O 1 s XPS spectra for the powder and thin film samples are shown in Figure 3(c). It can be observed that the original powder sample presents nonsymmetric O 1 s peak centered at 531.3 eV with a shoulder around 530 eV. The peak at 531.3 eV corresponds to the oxygen in the acetylacetonic ligand cycle^30^, while the shoulder at 530 eV should attribute to the oxygen bonded with zirconium^49^,^50^, which means there is a small portion (17% from the peak fitting) of Zr^4+^ ions coordinate less than four acetylacetonic ligands in the original power sample and this is a common case in coordination complexes^51^. The thin film sample shows similar O 1 s peak, and the portion of non-four-coordination is 15%, which means there is no decomposition occurred. As shown in Figure 3(d), the C 1 s peaks of ZrAcac powder are located at 284.7 eV, which is assigned to the carbon in C-H/C-C group^52^, the peak at 286.7 eV corresponds to the carbon in delocalized C = O of ligand cycle^52^, and the weak shoulder at 288.3 eV attributes to the absorbed CO_2_ from ambient atmosphere^52^. The atomic ratio of C 1 s (286.7 eV) and C 1 s (284.7 eV) peaks is 1:3.50, which is very close to the ratio of 1:3.54 for thin film sample. The XPS results confirm that there is no structure change between the powder and the obtained thin film.

**Figure 4:**
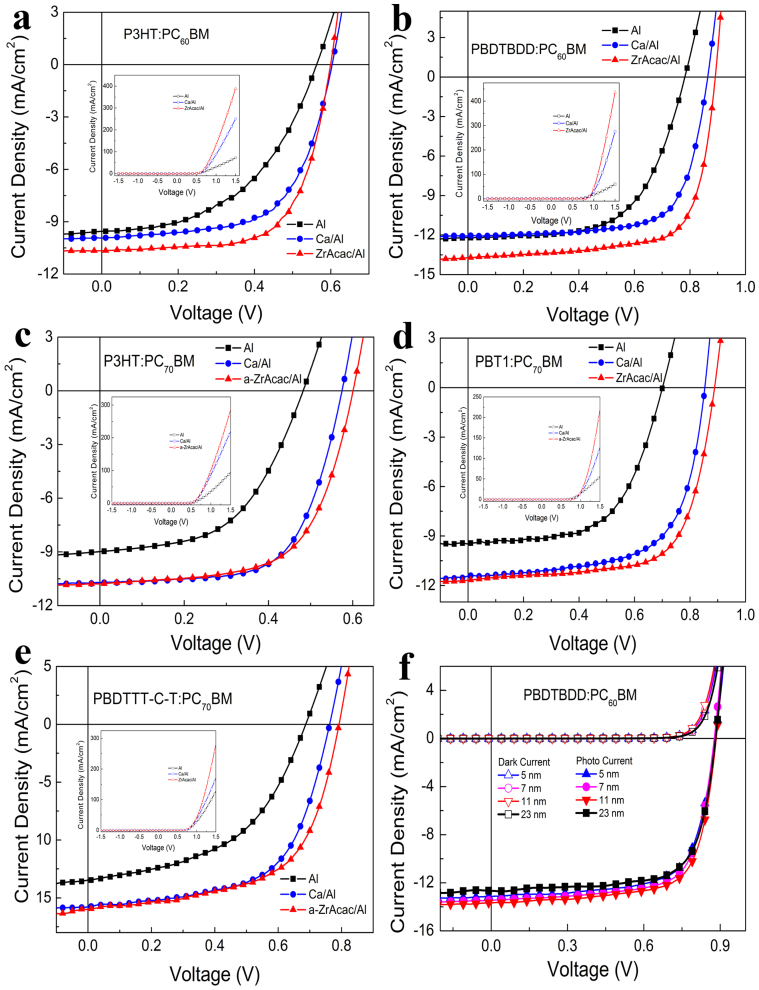
*J−V* curves under the illumination of AM 1.5 G, 100 mW/cm^2^ for the PSCs based on (a) P3HT:PC_60_BM, (b) PBDTBDD:PC_60_BM, (c) P3HT:PC_70_BM, (d) PBT1:PC_70_BM, and (e) PBDTTT-C-T:PC_70_BM with different cathode, insets are dark current at full bias scan from −1.5 V to 1.5 V; (f) *J−V* curves of the PSCs based on PBDTBDD:PC_60_BM with varied thickness of a-ZrAcac cathode buffer layer.

Figure 4 shows current density-voltage (*J*-*V*) curves of the PSCs based on P3HT:PC_60_BM, PBDTBDD:PC_60_BM, P3HT:PC_70_BM, PBT1:PC_70_BM and PBDTTT-C-T:PC_70_BM with different cathode in the dark and under the illumination of AM 1.5 G, 100 mW/cm^2^. The device performance parameters are given in Table 1, which are average values of 20 individual devices. For example, the *V*_oc_, *J*_sc_, FF and PCE for PBDTBDD:PC_60_BM based on 20 devices are listed in Table S1 in SI. As shown in the inset of Figure 4(a), the *J-V* curves measured in the dark for the PSCs based on P3HT:PC_60_BM show quite different charge injection and rectification behavior for the devices with different cathode. At positive bias of 1.5 V, the injection current of the device with Al cathode is 72.3 mA/cm^2^, with a rectification ratio of 8.03 × 10^3^ at ±1.5 V. While that of the device with Ca/Al as cathode increased to 250.2 mA/cm^2^ with a rectification ratio of 1.39 × 10^4^. Interestingly, when utilizing a-ZrAcac/Al as cathode, the injection current is dramatically increased to 388.8 mA/cm^2^ with a rectification ratio of 3.09 × 10^4^, which is even much higher than that of the Ca/Al based device. The ZrAcac CBL works very well in PBDTBDD:PC_60_BM based devices, the injection current at 1.5 V and rectification ratio at ±1.5 V for device with ZrAcac buffer layer (Figure 4(b) inset) reach 436.0 mA/cm^2^ and 9.91 × 10^3^, respectively, much higher than that of the PSCs with Ca/Al (275.9 mA/cm^2^, 2.39 × 10^3^) or Al (59.7 mA/cm^2^, 1.90 × 10^3^) as cathode. The enhanced charge injection and improved rectification ratio also can be seen in PC_70_BM based P3HT (Figure 4(c)) and low bandgap PBT1 (Figure 4(d)) and PBDTTT-C-T (Figure 4(e)) devices with a-ZrAcac CBL.

The PSCs based on P3HT:PC_60_BM with only Al cathode gives a PCE of 2.65%, with an open-circuit voltage (*V_oc_*) of 0.56 V, a short-circuit current density (*J_sc_*) of 9.55 mA/cm^2^, and a fill factor (FF) of 49.6%. In contrast, the four parameters of *V_oc_*, *J_sc_*, FF, and PCE for the device with a-ZrAcac/Al cathode are all enhanced to 0.60 V, 10.66 mA/cm^2^, 66.1%, and 4.23%, respectively. The photovoltaic performance of the device with a-ZrAcac/Al cathode is also significantly improved than that of the device with traditional Ca/Al cathode with PCE increased by 12.8%. Furthermore, the a-ZrAcac CBL shows excellent performance in the PSCs based on low bandgap D-A copolymer PBDTBDD:PC_60_BM, as shown in Figure 4(b) and Table 1. The control device with only Al cathode shows a PCE of 5.72%, with a *V_oc_* of 0.78 V, a *J_sc_* of 12.10 mA/cm^2^, and an FF of 60.6%. With Ca/Al cathode, a similar *J_sc_* is obtained, while both the *V_oc_* and the FF are improved to 0.86 V and 70.8%, respectively, achieving an enhanced PCE of 7.34%. Surprisingly, the *V_oc_*, *J_sc_* and PCE of the PSCs with a-ZrAcac/Al cathode are all greatly enhanced to 0.88 V, 14.28 mA/cm^2^ and 8.75%, respectively. The best device shows a PCE of 9.23%, a *V*_oc_ of 0.89 V, a *J*_sc_ of 14.25 mA/cm^2^, and a FF of 72.7%, among the highest values reported in the literature so far for PSCs. For the significantly increased *V*_oc_ of the devices with Ca/Al or a-ZrAcac/Al cathode in comparison with that with Al cathode, it should be ascribed to the lower work function of Ca and ZrAcac compared with that of Al. In comparing the a-ZrAcac/Al based devices with the Ca/Al based devices, the improved PCE is mainly attributed to the enhanced *J_sc_* and FF. The increased FF for the devices with a-ZrAcac/Al cathode could result from the enhanced charge extraction, increased light harvest and greatly decreased series resistance^44^ (see Table 1). The series resistance (*R_s_*) of the PSCs based on P3HT:PC_60_BM is decreased from 13.0 ohm for Al cathode to 3.5 ohm for Ca/Al cathode and to 2.3 ohm for the a-ZrAcac/Al cathode, and that of the PSCs based on PBDTBDD:PC_60_BM is decreased from 11.5 ohm for Al cathode to 2.0 ohm for Ca/Al cathode and to 1.2 ohm for the a-ZrAcac/Al cathode.

The a-ZrAcac CBL is of great compatibility with a variety of photoactive layers and shows good photovoltaic performance. As shown in Figure 4(c–e), with modified by a-ZrAcac layer, the PCE of the devices based on P3HT:PC_70_BM, PBT1:PC_70_BM, and PBDTTT-C-T:PC_70_BM reaches 4.01%, 7.06%, and 7.55%, respectively, increased by 42%, 80%, and 66%, in comparison with the devices with bare Al cathode. It's also higher than that (3.88%, 6.34% and 7.22%, respectively) of the devices with Ca/Al electrodes. Comparing the performance of PC_70_BM based devices, the enhanced PCE for a-ZrAcac buffered devices can mainly attributed to the enhanced *V_oc_*, since the *J*_sc_ and FF is similar to the devices with Ca/Al cathodes. The improved *V*_oc_ should attribute to the interfacial dipoles^47^ formed by inserting a-ZrAcac layer as shown in Figure 1(c).

The *J-V* curves of PBDTBDD:PC_60_BM and P3HT:PC_60_BM based devices with varied a-ZrAcac thickness are shown in Figure 4(f) and Figure S6, respectively, and the parameters of the devices are summarized in Table S2 and Table S3 in SI, respectively. The *J*_sc_ is insensitive to a-ZrAcac layer thickness in a wide thickness range, while the overall performance critically depends on its thickness, because a too thick interfacial layer will induce a high series resistance, while a too-thin layer could not provide an ohmic contact for electron extraction.

## Discussion

**Figure 5:**
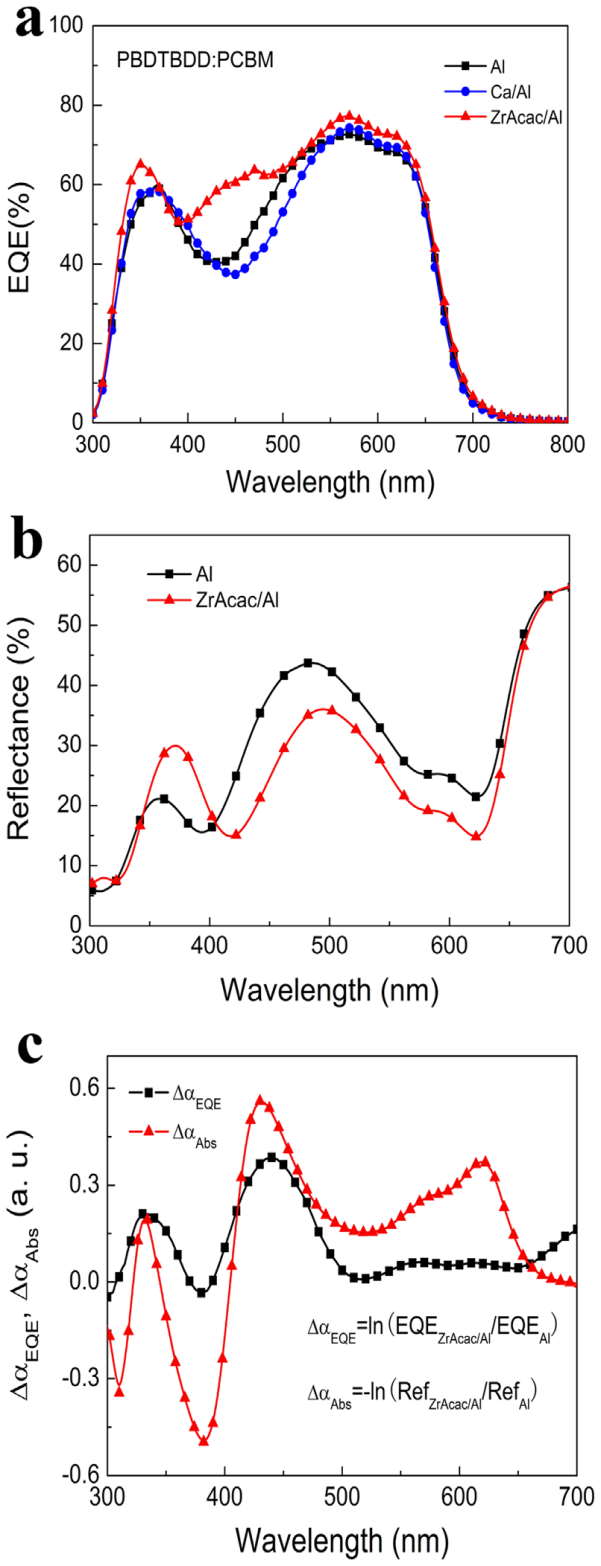
(a) External quantum efficiency (EQE) spectra, and (b) the reflectance spectra of the PSCs based on PBDTBDD:PC_60_BM with different cathode; (c) the additional absorption spectrum as well as additional EQE spectrum calculated from the difference of reflectance spectra and EQE spectra of the PSCs based on PBDTBDD:PC_60_BM with a-Zracac/Al and Al cathode, respectively.

In order to further elucidate the increased *J_sc_* for the devices with a-ZrAcac CBL, we compared the external quantum efficiency (EQE) spectra of the PSCs with Al, Ca/Al and a-ZrAcac/Al cathodes, as given in Figure 5(a) (PBDTBDD:PC_60_BM based devices) and Figure S7 (P3HT:PC_60_BM based devices) in SI. As shown in Figure 5(a), in comparison with the devices with Al and Ca/Al cathode, the EQE spectra of PSCs based on PBDTBDD:PCBM with a-ZrAcac CBL exhibits enhanced light response in the whole absorption band of 300–700 nm, giving a notable enhancement in wavelength range from 380 nm to 500 nm, where it is a deep valley for the devices with Al and Ca/Al cathode due to the limited absorption of the polymer as shown in Figure S8 in SI.

To further clarify the different light response behavior, we measured the reflectance spectra of the devices with Al and a-ZrAcac/Al cathode. As shown in Figure 5(b), the reflectance of the device with a-ZrAcac/Al cathode is lower than that of the device with only Al cathode in the wavelength range of 400–650 nm. Actually, the highly transparent characteristic and appropriate thickness of the a-ZrAcac layer could be its advantage on the reflectance over Ca or other organic CBL. To investigate the relationship between the enhanced light harvest (reduced light reflectance) and the increased EQE values (photocurrent response), we derived the additional absorption, Δ*α*_A*bs*_, and the additional EQE, Δ*α*_EQE_, from the reflectance and the EQE spectra, respectively, where Δ*α_Abs_* = −ln(Ref*_Zracac_*_/*Al*_/Ref*_Al_*), and Δ*α_EQE_* = −ln(*EQE_Zracac_*_/*Al*_/*EQE_Al_*. Figure 5(c) shows the Δ*α*_A*bs*_ and Δ*α*_EQE_ curves as the function of wavelength. It can be seen that the shape of Δ*α*_EQE_ curve is very similar to that of the Δ*α*_A*bs*_ curve, indicating that the enhanced photocurrent in the device with a-ZrAcac/Al cathode can be mainly ascribed to the additional absorption of the photoactive layer in the device.

**Figure 6:**
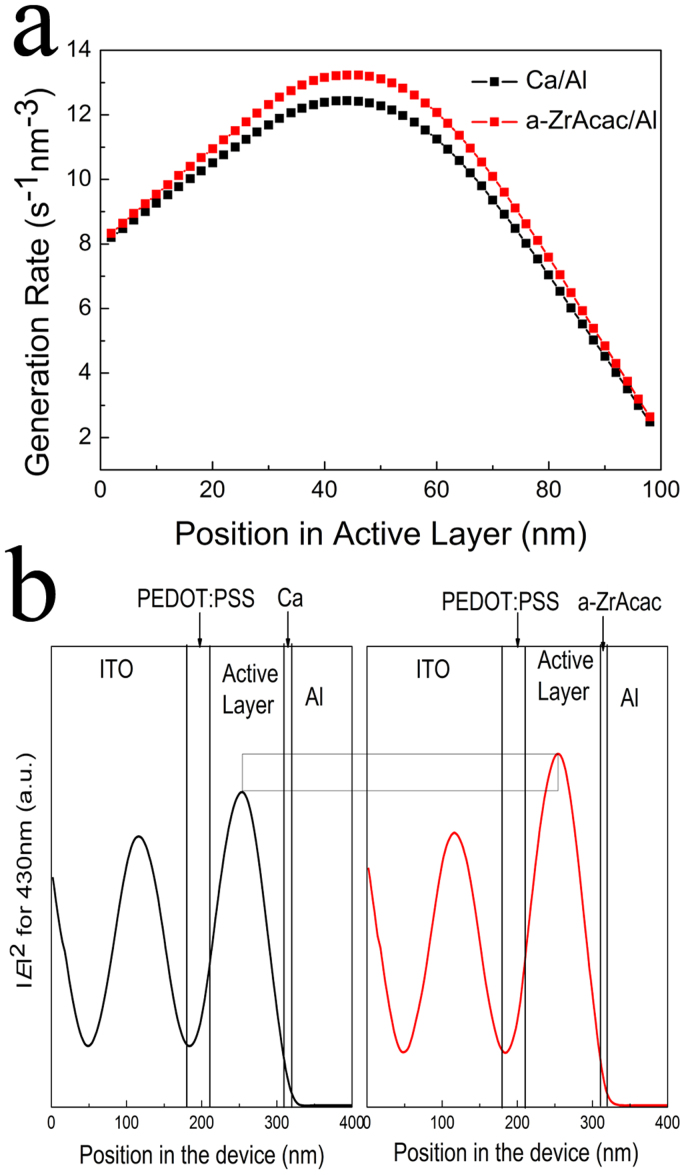
(a) Modeled exciton generation rate in the PBDTBDD:PC_60_BM active layer (100 nm) as a function of position within the active layer with Ca/Al and a-ZrAcac/Al cathode. (b) Calculated distribution of the normalized modulus squared of the optical electric field |E^2^| inside a photovoltaic device: ITO (~180 nm)/PEDOT (~30 nm)/PBDTBDD:PC_60_BM (~100 nm)/Ca (10 nm) or a-ZrAcac (11 nm)/Al (90 nm) for a wavelength of 430 nm.

To gain further insight into the origin of the enhanced *J*_sc_ in the device, one-dimensional transfer matrix formalism^53^ based on optical modelling calculations was conducted, where experimentally measured refractive indices and extinction coefficients were used. The exciton generation rate versus position in the PBDTBDD:PC_60_BM active layer (100 nm) is shown in Figure 6(a) for devices with Ca/Al and a-ZrAcac/Al cathode, verifying that the device with a-ZrAcac/Al cathode can generate more carriers than that of the Ca/Al based device, which accounts in part for the higher *J*_sc_ of the device with a-ZrAcac/Al cathode. The calculated exciton generation rate is in good agreement with EQE measurement as shown in Figure 5(a). The normalized modulus of the optical electric field is shown in Figure 6(b) for the PSCs with an active layer thickness of 100 nm and with Ca/Al and a-ZrAcac/Al cathode for a wavelength of 430 nm. This wavelength has been chosen as the difference in EQE, and absorption between the two device stacks is most prominent at 430 nm. Modified with a-ZrAcac causes little shift of the maximum of the electric field but greatly increases the overall modulus throughout the whole active layer. The calculated results of enhanced light distribution within the active layer for a-ZrAcac based device is in agreement with tested reflection spectra as shown in Figure 5(b).

**Figure 7:**
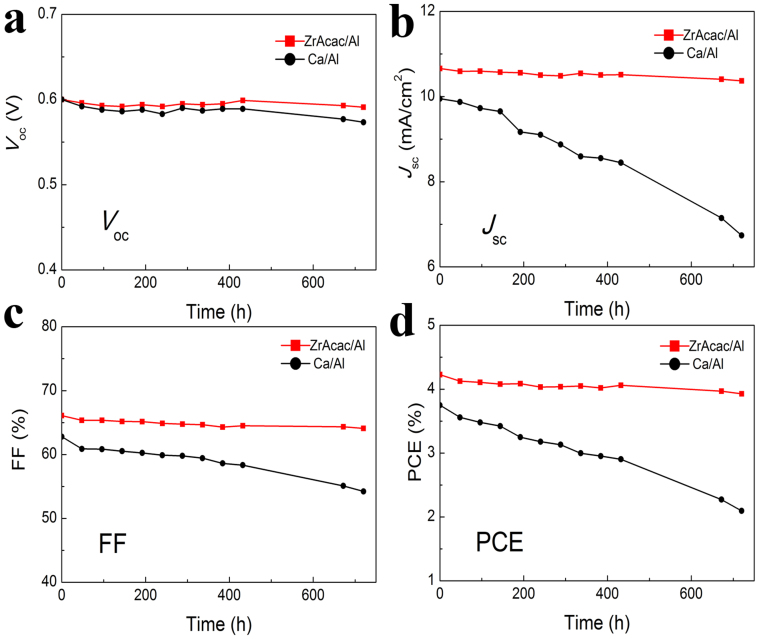
Long-term stability of the P3HT:PC_60_BM based PSCs with Ca/Al or a-ZrAcac/Al cathode. The evolution of (a) *V*_oc_, (b) *J*_sc_, (c) FF, and (d) PCE with time. The devices were stored in an N_2_ filled glove-box.

The long term stability of non-encapsulated P3HT:PC_60_BM based PSCs with a-ZrAcac/Al and Ca/Al cathodes were investigated by testing the evolution of the characteristic parameters (*V*_oc_, *J*_sc_, FF, and PCE) with time in a nitrogen filled glove-box (the concentration of H_2_O and O_2_ is less than 20 ppm) as shown in Figure 7. After 720 hours (30 days), the PCEs of the devices with Ca/Al drop to about 56% of the initial PCEs. The observed decrease in the PCE is mainly due to a deterioration of both *J*_sc_ and FF, with a little drop (5%) of *V*_oc_. On the contrary, the devices with a-ZrAcac/Al cathodes were found to show dramatically improved stability and after 30 days retained 93% of its initial PCE under N_2_. This is in excellent agreement with the stability of PSCs using solution processed PFN layers reported previously^32^. These results indicate that the a-ZrAcac interfacial layer can effectively improve the long term stability of PSCs.

In summary, we successfully demonstrate high performance PSCs by employing as-prepared ZrAcac film spin-cast from its ethanol solution as CBL. The devices with a-ZrAcac/Al cathode show decreased series resistance and enhanced photocurrent due to the remarkable improvement of the charge extraction and light harvesting. The PCE of the device based on P3HT:PC_60_BM with a-ZrAcac CBL reaches 4.23% under the illumination of AM1.5G 100 mW/cm^2^. When choosing D-A copolymer PBDTBDD as electron donor, an average PCE of 8.75% and a best PCE of 9.23% was achieved, which is greatly improved (19% enhancement) in comparison with that (7.34%) of the device with traditional Ca/Al cathode. Our findings indicate that a-ZrAcac is a promising CBL for the fabrication of large area flexible PSCs.

## Methods

### Device fabrication and characterization

The ITO glass (sheet resistance:10 Ω/sq, CSG Holding, China) was sequential ultrasonic cleaned in detergent, deionized water, acetone, and isopropanol, and then treated in an ultraviolet-ozone chamber (Jelight, USA) for 20 min. PEDOT:PSS (Clevious P VP AI 4083, H. C. Stark) aqueous solution was filtered through a 0.45 μm filter and spin-coated at 2000 rpm for 60 s on the pre-treated ITO substrate, followed by 150°C baking for 10 min in air, achieving a 30 nm thick PEDOT:PSS layer. Subsequently, the PEDOT:PSS modified substrate was transferred to a nitrogen-filled glove-box for photoactive layer and cathode buffer layer preparation. The P3HT (Rieke Metals) and PC_60_BM or PC_70_BM (Nano-C) blend layer was prepared by spin-coating (800 rpm) the 1, 2-dichlorobenzene (ODCB) solution of P3HT and PC_60_BM or PC_70_BM (1:1 w/w, polymer concentration of 20 mg/mL) on the PEDOT:PSS modified ITO electrode for 20 s, followed by solvent-annealing^54^ in covered glass Petri dish, obtaining a 240 nm thick photoactive layer. The PBDTBDD and PC_60_BM blend photoactive layer was prepared by spin-coating (1200 rpm) the dichlorobenzene solution of the mixed solution (1:1 w/w, polymer concentration of 15 mg/mL) with 3% volume ratio of 1,8-Diiodooctane (DIO, Sigma Aldrich) on the PEDOT:PSS modified ITO electrode. The thickness of the photoactive layer is about 100 nm. The PBT1:PC_70_BM blend photoactive layer was prepared by spin-coating (800 rpm) the ODCB solution of the mixed solution (1:1 w/w, polymer concentration of 10 mg/mL) with 1% volume ratio of DIO on the PEDOT:PSS modified ITO electrode for 60 s, obtaining a ca. 80 nm thick film. The PBDTTT-C-T:PC_70_BM blend film was fabricated by spin-coating (1500 rpm) the ODCB solution of the mixed solution (1:1.5 w/w, polymer concentration of 12.5 mg/mL) with 3 vol% DIO on the ITO/PEDOT:PSS electrode for 60 s, yielding a ca. 80 nm thick photoactive layer. The ZrAcac powder was bought from Alfa Aesar with a purity of 98%. The ZrAcac ethanol solution was simply made by adding the ZrAcac powder into the anhydrous ethanol solvent then stirred for 3 h, obtaining a colorless solution with concentration of 0.25–2 mg/mL. The ZrAcac cathode buffer layer was prepared by directly spin-coating (1500–6000 rpm) the ZrAcac ethanol solution on the photoactive layer for 60 s to obtain the film with different thickness, no any additional thermal annealing or post-treatment was performed. The just prepared ZrAcac thin layer named as a-ZrAcac layer. Finally, the substrate was transferred to a vacuum chamber and the metal cathode (Al or Ca/Al) was thermally deposited. The photoactive area of the device is around 4 mm^2^ defined by perpendicular ITO and cathode electrode. The current density–voltage (*J–V*) and the external quantum efficiency measurements were conducted according to our previous publication^30^.

### Instrumentation

The surface morphologies of the P3HT:PC_60_BM and PBDTBDD:PC_60_BM photoactive layers with and without ZrAcac layer were analyzed using a VEECO DICP-II atomic force microscope (AFM) operated in the tapping mode under ambient atmosphere at room temperature. The reflectance spectra were measured by LAMBDA 950 UV/Vis/NIR Spectrophotometer with the device structures of ITO/PEDOT:PSS/PBDTBDD:PC_60_BM/Al and ITO/PEDOT:PSS/PBDTBDD:PC_60_BM/a-ZrAcac/Al. The thickness of the photoactive layer was measured by Ambios Technology XP-2 surface profilometer. An ESCA Lab220i-XL electron spectrometer from VG Scientific using 300 W Al *K* α radiation operated at a base pressure of 3 × 10^−9^ mbar was used to obtain XPS data. The binding energies were referenced to adventitious carbon (C 1 s line at 284.8 eV). The ultraviolet photoelectron spectroscopy (UPS) measurements were conducted in ultra-high vacuum (3.0 × 10^−8^ Torr) with a Kratos Axis Ultra DLD ultraviolet photoelectron spectrometer equipped with a monochromatic He ultraviolet source He I (*hν* = 21.22 eV). To separate the sample and the secondary edge for the analyzer, a sample bias of −9 V was applied. The optical constants of a-ZrAcac and the photoactive blend were derived from spectroscopic ellipsometry by SENTECH SE400 rotating compensator ellipsometer.

## Author Contributions

Z.A.T., S.S.L., J.L. and Y.F.L. designed, fabricated and characterized the devices. D.P.Q. and J.H.H. synthesized the polymer PBDTBDD. Z.A.T. and F.Z.W. conducted the optical simulation. All authors discussed the results, and Z.A.T., S.S.L. and Y.F.L. write the manuscript.

## Supplementary Material

Supplementary Information Supporting Information

## Figures and Tables

**Table 1:** Device parameters of the PSCs based on P3HT:PC_60_BM, PBDTBDD:PC_60_BM, P3HT:PC_70_BM, PBT1:PC_70_BM and PBDTTT-C-T:PC_70_BM with different cathode under the illumination of AM 1.5 G, 100 mW/cm^2^

Active Layer	Cathode	*V*_oc_ (V)	*J*_sc_ (mA/cm^2^)	FF (%)	PCE (%)	*R*_s_[a] (Ω cm^2^)
P3HT:PC_60_BM	Al	0.56	9.55	49.6	2.65	13.0
	Ca/Al	0.60	9.95	62.8	3.75	3.5
	a-ZrAcac/Al	0.60	10.66	66.1	4.23	2.3
PBDTBDD:PC_60_BM	Al	0.78	12.10	60.6	5.72	11.5
	Ca/Al	0.86	12.06	70.8	7.34	2.0
	a-ZrAcac/Al	0.88	14.28	69.6	8.75	1.2
P3HT:PC_70_BM	Al	0.55	9.11	56.5	2.83	8.9
	Ca/Al	0.58	10.71	62.5	3.88	3.8
	a-ZrAcac/Al	0.60	10.73	62.3	4.01	2.8
PBT1:PC_70_BM	Al	0.70	9.50	58.9	3.92	12.4
	Ca/Al	0.86	11.44	64.4	6.34	5.1
	a-ZrAcac/Al	0.88	11.68	68.7	7.06	3.2
PBDTTT-C-T:PC_70_BM	Al	0.68	13.47	49.7	4.55	5.4
	Ca/Al	0.76	15.76	60.3	7.22	4.0
	a-ZrAcac/Al	0.79	15.95	60.0	7.55	2.4

^[a]^Series resistance (*R_s_*) of the PSCs in the dark are obtained at around 1.2 V.
