# Supplementary material for: High performance polymer solar cells with as-prepared zirconium acetylacetonate film as cathode buffer layer
Source: Sci Rep. 2014 Apr 15;4:4691. doi: 10.1038/srep04691 (PMC3986729; doi:10.1038/srep04691)
Supplement: Supplementary Information — Supporting Information [file srep04691-s1.doc]

**Supporting Information**

**High performance polymer solar cells with as-prepared zirconium acetylacetonate film as cathode buffer layer**

Zhan’ao Tan^1^,*, Shusheng Li^1^, Fuzhi Wang^1^, Deping Qian^1^, Jun Lin^1^, Jianhui Hou^2^, and Yongfang Li^2^,*

The Absorption spectra of ZrAcac layer spin-coating on quartz glass from 0.5 mg/mL ethanol solution at 3000 rpm for 30 s was measured by Hitachi U-3010 UV-Vis spectrophotometer. The thermogravimetric analysis (TGA) and differential thermal analysis (DTA) were conducted on a Perkin–Elmer 7 thermogravimetric analyzer with a heating rate of 20 ℃/min under ambient atmosphere. XRD patterns were obtained with a Siemens D5005 diffractometer using Cu K*a* radiation at 40 kV and 20 mA.


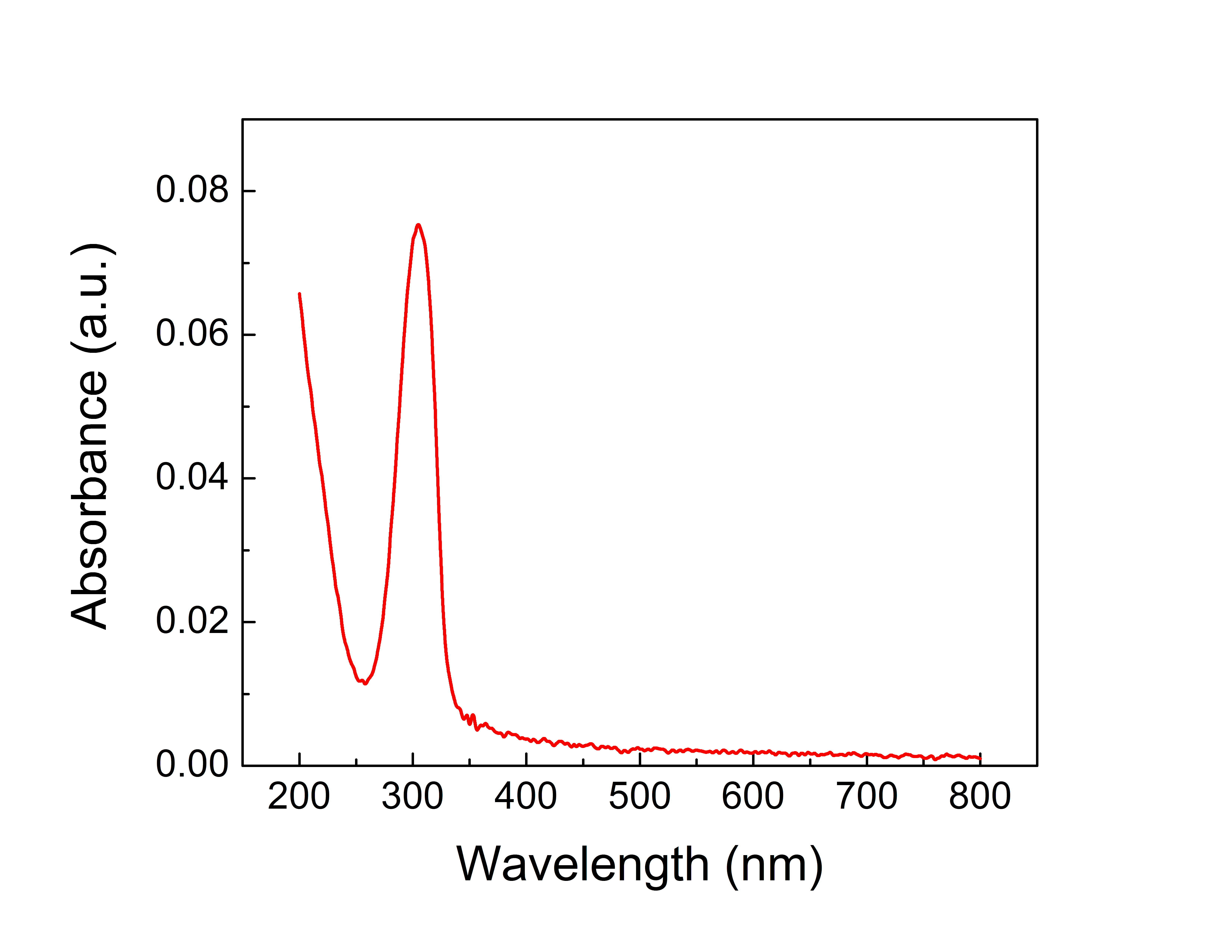


**Figure S1**. Absorption spectra of ZrAcac layer spin-coating on quartz glass from 0.5mg/mL ethanol solution at 3000 rpm for 30 s.


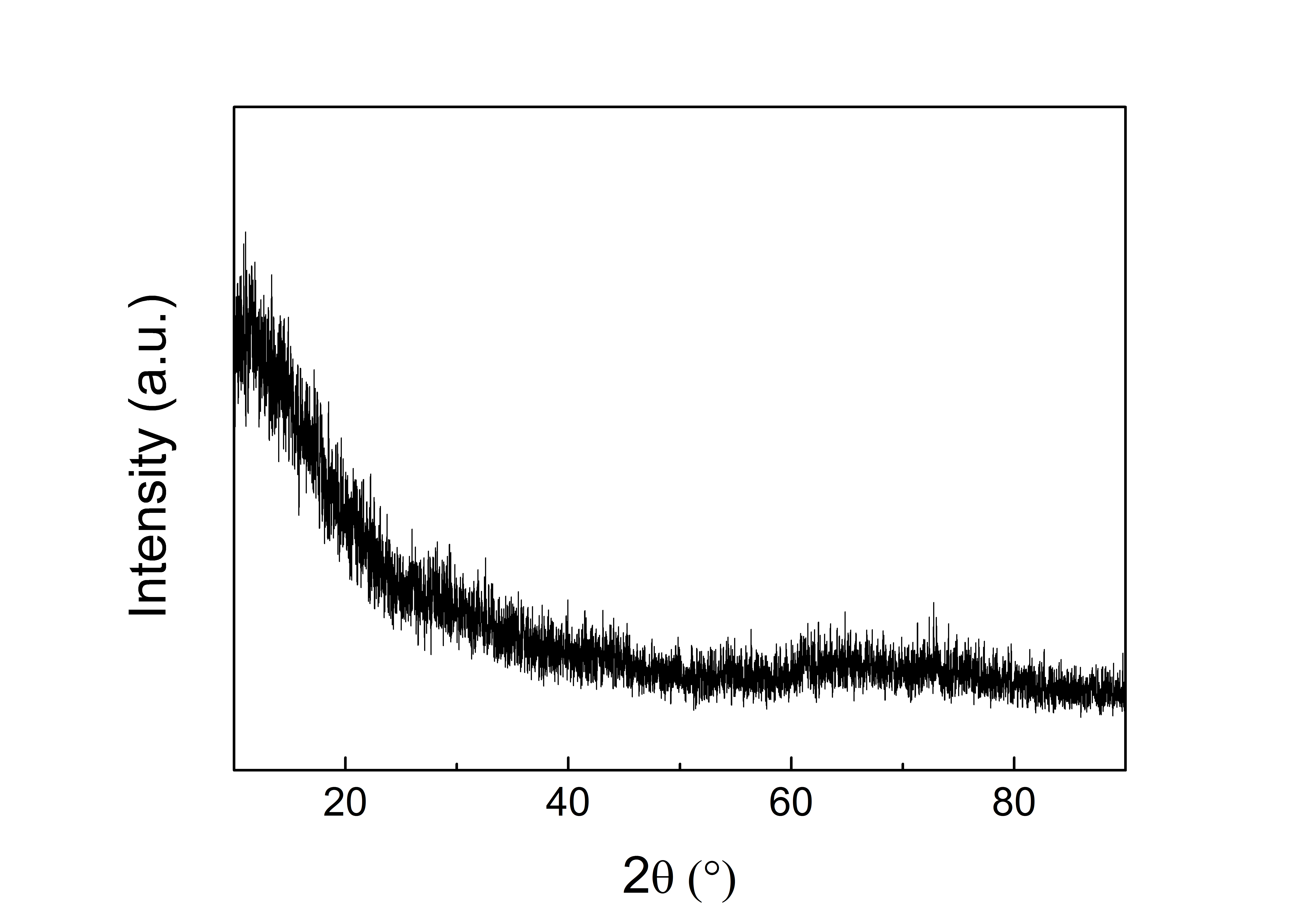


**Figure S2**. X-ray diffraction (XRD) spectra of a-ZrAcac layer (30 nm) spin-coated on the clean silicon substrate surface.


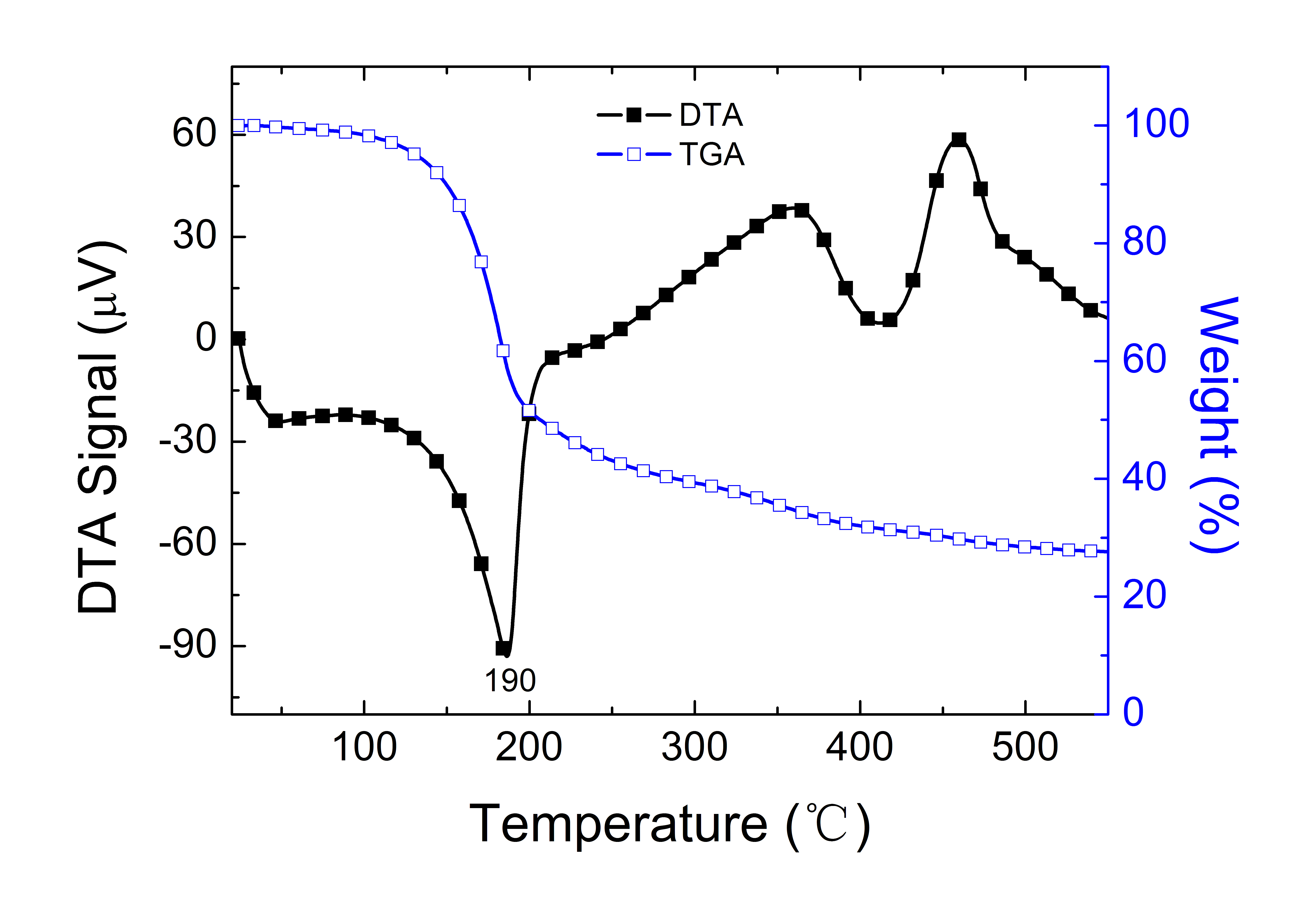


**Figure S3**. Thermogravimetric and differential thermal analysis of ZrAcac powder in N_2_.


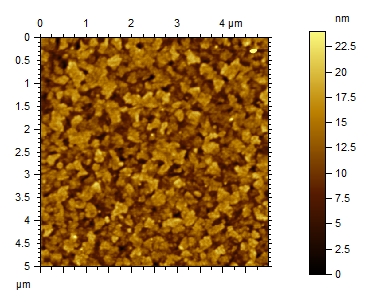


**Figure S4**. AFM image of the ZrAcac layer spin-coated on clean ITO substrate.


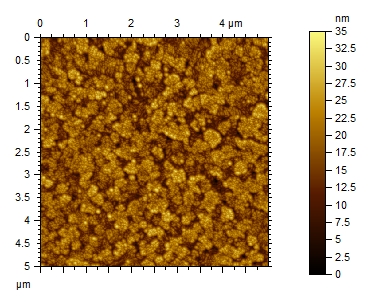


**Figure S5**. AFM image of the bare ITO substrate.

**Table S1**. Device parameter (*V*_oc_, *J*_sc_, FF and PCE) variation of 20 individual pieces of PBDTBDD:PC_60_BM based devices with structure of ITO/PEDOT:PSS (30 nm)/PBDTBDD:PC_60_BM (100 nm) /a-ZrAcac (11 nm)/Al (100 nm).

| Number | *V*_oc_ (V) | *J*_sc_ (mA/cm^2^) | FF (%) | PCE (%) |
| --- | --- | --- | --- | --- |
| 1 | 0.88 | 14.48 | 68.2 | 8.68 |
| 2 | 0.88 | 15.19 | 67.8 | 9.06 |
| 3 | 0.89 | 13.63 | 70.9 | 8.59 |
| 4 | 0.89 | 14.25 | 72.7 | 9.23 |
| 5 | 0.89 | 14.29 | 72.2 | 9.19 |
| 6 | 0.88 | 14.13 | 72.4 | 9.01 |
| 7 | 0.88 | 14.93 | 67.1 | 8.82 |
| 8 | 0.87 | 15.28 | 67.1 | 8.92 |
| 9 | 0.88 | 14.26 | 68.9 | 8.64 |
| 10 | 0.87 | 14.95 | 67.3 | 8.76 |
| 11 | 0.87 | 15.09 | 65.8 | 8.64 |
| 12 | 0.88 | 14.43 | 67.8 | 8.61 |
| 13 | 0.89 | 13.54 | 72.7 | 8.77 |
| 14 | 0.89 | 13.57 | 72.2 | 8.73 |
| 15 | 0.88 | 13.42 | 72.4 | 8.56 |
| 16 | 0.88 | 14.19 | 68.1 | 8.50 |
| 17 | 0.87 | 14.52 | 67.1 | 8.48 |
| 18 | 0.88 | 13.60 | 71.2 | 8.52 |
| 19 | 0.88 | 13.41 | 71.6 | 8.45 |
| 20 | 0.88 | 14.43 | 67.8 | 8.61 |
| Average | 0.88 | 14.28 | 69.6 | 8.75 |

**Table S2**. The dependence of the photovoltaic performance of the PSCs based on PBDTBDD:PC_60_BM on the thickness of the a-ZrAcac CBL (deduced from Figure 2f)

| a-ZrAcac Thickness | *V*_oc_ (V) | *J*_sc_ (mA/cm^2^) | FF (%) | PCE (%) |
| --- | --- | --- | --- | --- |
| 5 nm | 0.89 | 12.95 | 70.9 | 8.16 |
| 7 nm | 0.89 | 13.26 | 71.6 | 8.45 |
| 11 nm | 0.89 | 13.54 | 72.7 | 8.77 |
| 23 nm | 0.89 | 12.62 | 71.3 | 8.01 |


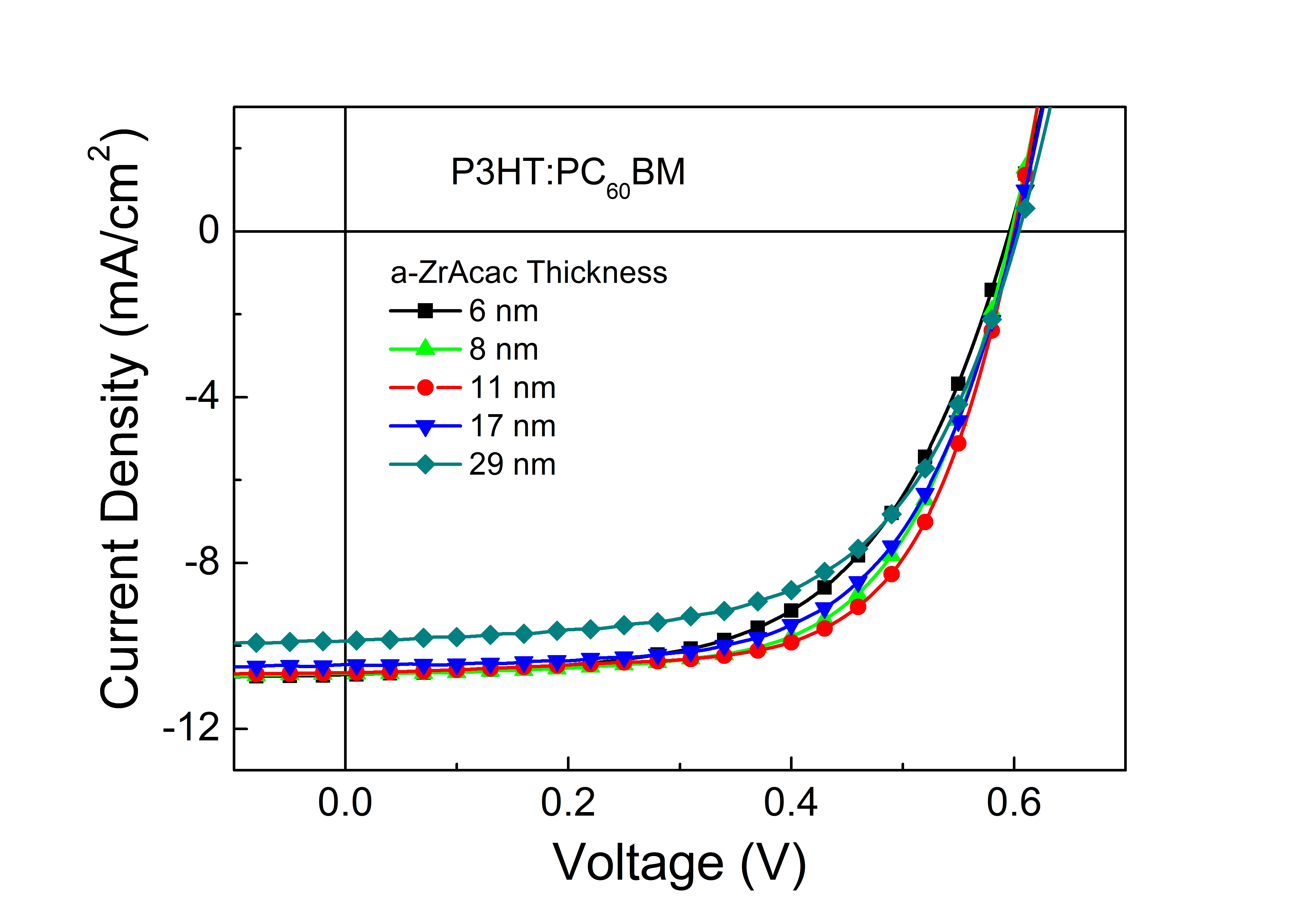


**Figure S6**. The experimental *J*–V characteristics of the PSCs with configuration of ITO/PEDOT:PSS (30 nm) /P3HT:PC_60_BM (240 nm)/a-ZrAcac (6, 8, 11, 17 or 29 nm)/Al (100 nm) under the illumination of AM 1.5G, 100 mW/cm^2^.

**Table S3**. The dependence of the photovoltaic performance of the PSCs based on P3HT:PC_60_BM on the thickness of the a-ZrAcac CBL (deduced from Figure S5)

| a-ZrAcac Thickness | *V*_oc_ (V) | *J*_sc_ (mA/cm^2^) | FF (%) | PCE (%) |
| --- | --- | --- | --- | --- |
| 6 nm | 0.59 | 10.70 | 58.6 | 3.70 |
| 8 nm | 0.59 | 10.67 | 64.2 | 4.04 |
| 11 nm | 0.60 | 10.66 | 66.1 | 4.23 |
| 17 nm | 0.60 | 10.46 | 62.5 | 3.92 |
| 29 nm | 0.60 | 9.89 | 59.7 | 3.54 |


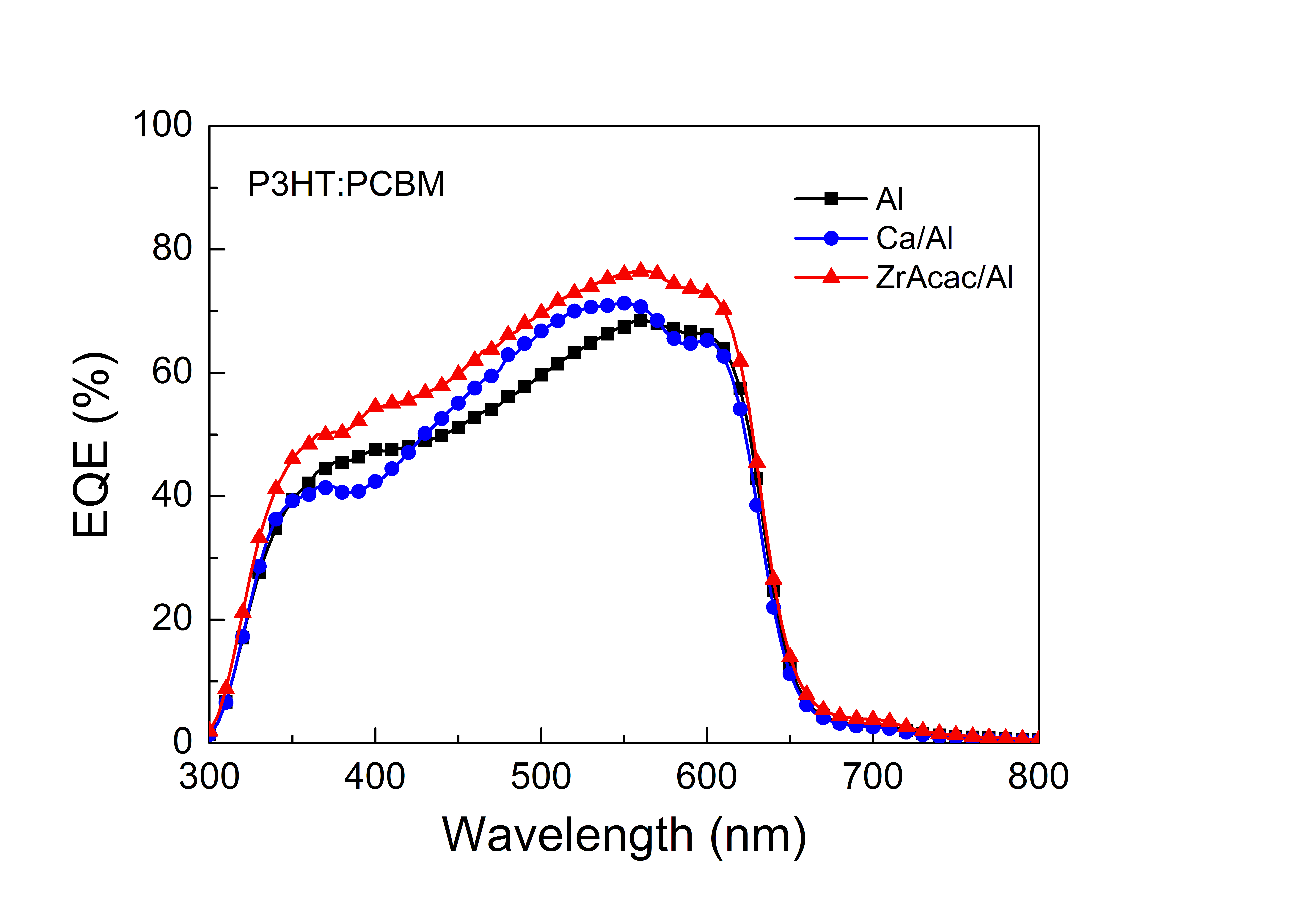


**Figure S7**. External quantum efficiency spectra of the PSCs based on P3HT:PC_60_BM with Al, Ca/Al and a-ZrAcac/Al cathode.


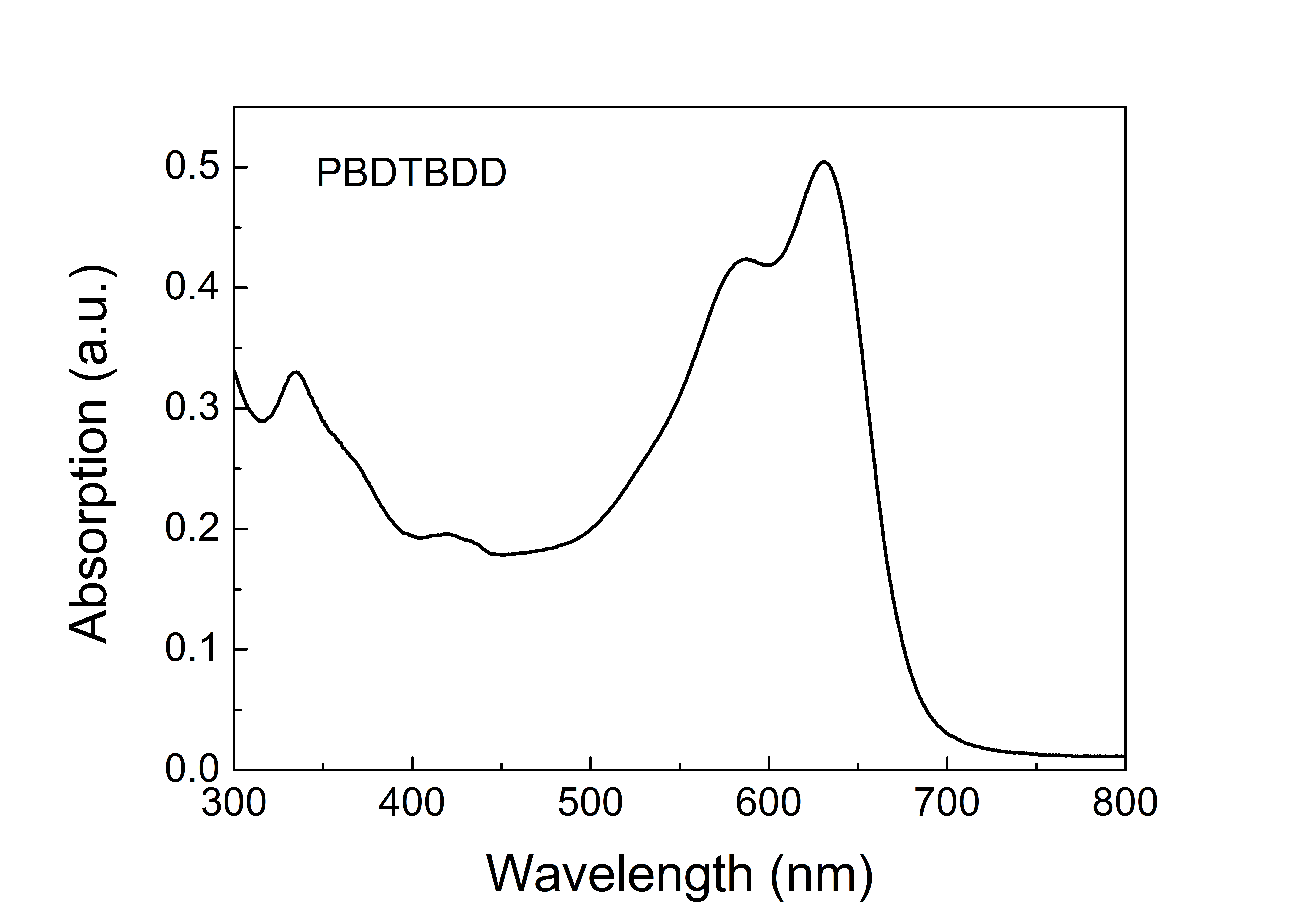


**Figure S8**. Absorption spectra of PBDTBDD thin film on quartz glass.
